# Efficient Mitochondrial Genome Editing by CRISPR/Cas9

**DOI:** 10.1155/2015/305716

**Published:** 2015-09-10

**Authors:** Areum Jo, Sangwoo Ham, Gum Hwa Lee, Yun-Il Lee, SangSeong Kim, Yun-Song Lee, Joo-Ho Shin, Yunjong Lee

**Affiliations:** ^1^Division of Pharmacology, Department of Molecular Cell Biology, Samsung Biomedical Research Institute, Sungkyunkwan University School of Medicine, Suwon, Gyeonggi-do 440-746, Republic of Korea; ^2^College of Pharmacy, Chosun University, Gwangju 501-759, Republic of Korea; ^3^Well Aging Research Center, Samsung Advanced Institute of Technology (SAIT), Yongin-si 446-712, Republic of Korea; ^4^Department of Pharmacy, Hanyang University, ERICA Campus, 55 Hanyangdaehak-ro, Sannok-gu, Ansan, Gyeonggi-do 426-791, Republic of Korea

## Abstract

The Clustered Regularly Interspaced Short Palindromic Repeats (CRISPR)/Cas9 system has been widely used for nuclear DNA editing to generate mutations or correct specific disease alleles. Despite its flexible application, it has not been determined if CRISPR/Cas9, originally identified as a bacterial defense system against virus, can be targeted to mitochondria for mtDNA editing. Here, we show that regular FLAG-Cas9 can localize to mitochondria to edit mitochondrial DNA with sgRNAs targeting specific loci of the mitochondrial genome. Expression of FLAG-Cas9 together with gRNA targeting Cox1 and Cox3 leads to cleavage of the specific mtDNA loci. In addition, we observed disruption of mitochondrial protein homeostasis following mtDNA truncation or cleavage by CRISPR/Cas9. To overcome nonspecific distribution of FLAG-Cas9, we also created a mitochondria-targeted Cas9 (mitoCas9). This new version of Cas9 localizes only to mitochondria; together with expression of gRNA targeting mtDNA, there is specific cleavage of mtDNA. MitoCas9-induced reduction of mtDNA and its transcription leads to mitochondrial membrane potential disruption and cell growth inhibition. This mitoCas9 could be applied to edit mtDNA together with gRNA expression vectors without affecting genomic DNA. In this brief study, we demonstrate that mtDNA editing is possible using CRISPR/Cas9. Moreover, our development of mitoCas9 with specific localization to the mitochondria should facilitate its application for mitochondrial genome editing.

## 1. Introduction

Mitochondria play roles in many important cellular functions including ATP production via oxidative phosphorylation, calcium storage, and regulation of cell death in various cell types [1–3]. Mitochondria contain their own genome, which encodes 13 proteins that are subunits of respiratory chain complexes, as well as two rRNAs and 22 mitochondrial tRNAs [4]. Due to the critical roles of genes encoded by mtDNA, maintenance of mitochondrial genome integrity is quite important for normal cellular functions. Mitochondrial DNA are, however, constantly under mutational pressure due to oxidative stress imposed by radicals generated by oxidative phosphorylation or an imbalance in the antioxidant defense system in aging or disease processes [1]. Damage to mtDNA, such as point mutations or deletions, contributes to or predisposes individuals to a variety of human diseases. Particularly, dysfunctional mitochondria have been implicated in several neurodegenerative diseases including Parkinson's disease [5, 6]. Indeed, several Parkinson's disease-associated genes exhibit pathological interaction with mitochondria [7].

In this respect, mitochondrial function is known to be dysregulated in some disease processes in cells or animals. For example, carbonyl cyanide m-chlorophenyl hydrazone (CCCP), 1-methyl-4-phenyl-1,2,3,6-tetrahydropyridine (MPTP), and rotenone have been widely used to impair the electron transport chain in order to produce mitophagy or oxidative stress-induced neurodegeneration [8, 9]. Since these chemical treatments lack genetic specificity, direct manipulation of mitochondrial DNA could have potential to generate genetic cell or animal models mimicking mitochondrial dysfunction. Moreover, once designed to target mutant mtDNA, selective correction of damaged mitochondrial DNA could provide an opportunity for therapeutic application. Indeed, TALEN has been reengineered to localize to mitochondria and specifically remove truncated dysfunctional mtDNA [10].

Despite the huge potential of mitoTALEN-mediated mtDNA editing, more user-friendly and efficient alternative methods are necessary to overcome difficulties in mtDNA modification either for correction of dysfunctional mtDNA or for producing dysfunctional mtDNA in order to create mitochondria-associated disease models.

Here we report a novel approach to generate mtDNA dysfunction with the CRISPR/Cas9 system. Cas9, widely used for genome editing, showed distribution to mitochondria as well as the nucleus. Expression of FLAG-Cas9 with gRNAs designed to target mtDNA resulted in cleavage of mtDNA and alterations in mitochondrial integrity as determined by Western blots for some mitochondrial proteins. Moreover, regular FLAG-Cas9 was modified to contain mitochondrial targeting sequence instead of nuclear localization sequence (NLS) in order to localize it to mitochondria (namely, mitoCas9). MitoCas9 robustly localized to mitochondria; together with gRNA targeting of mtDNA, specific cleavage of mtDNA was observed, demonstrating its functional application for mtDNA editing. These results together demonstrate the successful application of CRISPR/Cas9 in mitochondrial genome editing and suggest the possibility for* in vitro* and* in vivo* manipulation of mtDNA in a site-specific manner.

## 2. Materials and Methods

### 2.1. Reagent, Plasmids, and Antibodies

For primary antibodies, we used mouse antibody against PARP1 (cat# 556494, 1 : 1,000, BD Bioscience), mitochondrial marker antibody sampler kit (cat# 8674, 1 : 3,000, Cell Signaling, rabbit antibody to CoxIV, Cytochrome C, HSP60, PHB1, pyruvate dehydrogenase, SDHA, and mouse antibody to SOD1), mouse antibody against GAPDH (GT239, 1 : 5,000, GeneTex), mouse antibody against FLAG (clone M2, Sigma), rabbit antibody against PINK1 (cat# 100-494, 1 : 2,000, Novus Biologicals), and mouse antibody against UQCRC2 (cat# ab14745, 1 : 3,000, Abcam). For secondary antibodies, we used horseradish peroxidase- (HRP-) conjugated mouse antibody against FLAG (cat# A8592, 1 : 5,000, Sigma-Aldrich), HRP-conjugated antibody against HA (1 : 3,000, Roche), HRP-conjugated mouse antibody against *β*-actin (AC15, 1 : 10,000, Sigma-Aldrich), HRP-conjugated sheep antibody against mouse IgG (cat# RPN4301, 1 : 5,000, GE Healthcare), HRP-conjugated donkey antibody against rabbit IgG (cat# RPN4101, 1 : 5,000, GE Healthcare), Alexa Fluor 488-conjugated donkey antibody against mouse IgG (H+L) (cat# A21202, 1 : 1,000, Invitrogen), and Alexa Fluor 568-conjugated donkey antibody against rabbit IgG (cat# A10042, 1 : 1,000, Invitrogen). LentiCRISPR and lentiCRISPR-sgRNA-eGFP#2 constructs were purchased from Addgene. Oligonucleotides were ordered from and synthesized by Cosmo Genetech.

### 2.2. Construction of Mitochondria-Targeting Cas9 and gRNAs Expressing DNA

sgRNA targeting mtDNA was cloned into the lentiCRISPR construct according to the instructions posted on the Addgene website (deposited by Dr. Feng Zhang Lab). The oligonucleotides used for construction of each sgRNA are listed in Table 1. NCBI and Ensembl genome browser database were used to retrieve mitochondrial genome sequence. Sequence integrity was verified by sequencing. LentiCRISPR-EGFP sgRNA 2 construct was reengineered to mitoCas9. Briefly, a FLAG tag and NLS in the N-terminus of Cas9 were removed and replaced with the following sequences in order to achieve a mitochondrial targeting sequence and HA tag: ATGTCCGTCCTGACGCCGCTGCTGCTGCGGGGCTTGACAGGCTCGGCCCGGCGG CTCCCAGTGCCGCGCGCCAAGATCCATTCGTTG (MTS), which encodes a mitochondrial targeting sequence (MTS) from subunit VIII of human cytochrome c oxidase, and peptides MSVLTPLLLRGLTGSARRLPVPRAKIHSL and TACCCATACGACGTCCCAGACTACGCT, which encode HA peptide YPYDVPDYA. To obtain DNA expressing gRNA targeting Cox1 region without expression of FLAG-NLS-Cas9, U6 promoter, gRNA targeting sequence, scaffold, and termination signal were PCR-amplified with the following primers using lentiCRISPR-sgRNA-Cox1 constructs as PCR templates. LentiCRISPR-sgRNA-eGFP#2 was used as a template for control gRNA PCR. Forward primer was as follows: CAGAGAGGGCCTATTTCCCATG. Reverse primer was as follows: CTAGAATTCAAAAAAGCACCGACTC. PCR products were separated in an agarose gel and purified using the Qiagen gel extraction kit. Purified PCR products were used for transient transfection together with mitoCas9-expressing plasmid.

### 2.3. Cell Culture and Transfection

Human embryonic kidney (HEK-293T) cells were grown in DMEM containing 10% FBS (vol/vol) and antibiotics in a humidified 5% CO_2_/95% air atmosphere at 37°C. For transient transfection, cells were transfected with indicated target vectors using X-tremeGENE reagent (Roche) according to the manufacturer's instructions. To assess cell growth, HEK-293T cells were transfected with mitoCas9 and gRNA constructs targeting Cox1 and Cox3 or eGFP as control. 5 days following transfection, cells were passed onto 24-well plates and maintained in pyruvate-deficient complete media. Cell counts were determined by trypan blue staining followed by unbiased cell counting via EVE automatic cell counter (NanoEnTeK) at the indicated time points.

### 2.4. Subcellular Fractionation

HEK-293T cells were subcellular fractionated into cytosol, mitochondria, and nucleus using the Qproteome Mitochondria Isolation Kit (Qiagen), following the instructions in the manual. Cytosolic fractions were further concentrated with acetone precipitation. The purity of each fraction was validated with Western blots using antibodies to marker proteins for cytosolic (GAPDH, 2% of total cytosolic fraction was used), mitochondrial (SDHA, 20% of total mitochondria fraction was used), and nuclear (PARP1, 5% of total nuclear fraction was used) subcellular fractions.

### 2.5. Real-Time Quantitative PCR

Total genomic DNA and mitochondrial DNA were extracted using DirectPCR tail lysis buffer (Viagen) supplemented with proteinase K (Roche) using protocols slightly modified from the manufacturer's instructions. Two days or five days after transient transfection, HEK-293T cells were briefly washed with PBS and lysed in 100 *μ*L DirectPCR lysis buffer supplemented with 3 *μ*L proteinase K per each well (12-well culture). Proteinase K was inactivated by incubating samples at 80°C for 1 hr following incubation at 55°C for 2 hrs. DNA extract was diluted 100-fold and directly used for real-time quantitative PCR (Rotor-Gene Q, Qiagen). For cDNA synthesis, five days after transient transfection of mitoCas9 and corresponding gRNA constructs, total RNA was extracted using Qiagen RNeasy Mini Kit following manufacturer's instructions. Using total RNA as a template, cDNA was synthesized (iScript cDNA synthesis kit, Bio-Rad) for downstream real-time PCR application. Using the primers indicated in Table 2, mtDNA regions comprising each gene or mitochondria-encoded genes of interest were amplified. Primers for Cox1 and Cox3 were annealed to mtDNA sequences flanking the cleavage sites targeted by sgRNA-Cox1 and sgRNA-Cox3, respectively, in order to monitor specific cleavage of mtDNA. GAPDH was used as an internal loading control to amplify genomic DNA. RTQ PCR was performed using SYBR green master mix (QuantiFast SYBR Green PCR Kit, Qiagen) according to the manufacturer's instructions.

### 2.6. Western Blot

Total protein lysates were prepared by directly adding 2x sample buffer supplemented with *β*-mercaptoethanol (Bio-Rad) to HEK-293T cells briefly washed with ice-cold PBS. After boiling the samples for 5 minutes, they were separated on SDS-PAGE and transferred to nitrocellulose membrane for immunoblot experiments. The blotted nitrocellulose membrane was Ponceau (Sigma) stained to visualize even transfer of proteins. Immunoblotting was performed with an antibody of interest with chemiluminescence visualization (Pierce). The densitometric analyses of the bands were performed using ImageJ (NIH, http://rsb.info.nih.gov/ij/).

### 2.7. Immunofluorescence

HEK-293T cells, 24 hrs after transient transfection with the indicated constructs, fixed in 4% paraformaldehyde/PBS (pH 7.4) were blocked with 10% donkey serum (Sigma-Aldrich)/PBS plus 0.3% Triton X-100 and incubated with antibodies against FLAG or HA and CoxIV. For mitotracker Red labeling of functional mitochondria, cells were incubated with mitotracker Red CMXRos (200 nM, 10 min, Molecular Probes, Invitrogen) before PFA fixation. After brief washes with PBS, cells on glass plates were incubated with corresponding secondary antibodies conjugated with fluorescent dyes. After DAPI nuclear staining, samples were mounted with mounting solution (Aqua-Mount, Thermoscientific). Images were obtained using a fluorescent microscope (Zeiss Axiovert 200 M).

### 2.8. Statistics

Quantitative data are presented as mean ± s.e.m. Statistical significance was assessed via an unpaired two-tailed Student's *t*-test for comparison of two groups (control and test) or an ANOVA test and Student-Newman-Keuls post hoc analysis for comparison among multiple groups of more than three as indicated in each figure legend. *P* values lower than 0.05 were considered to indicate significant difference among groups.

## 3. Results

### 3.1. CRISPR/Cas9 Distribution to Mitochondria

**Figure 1:**
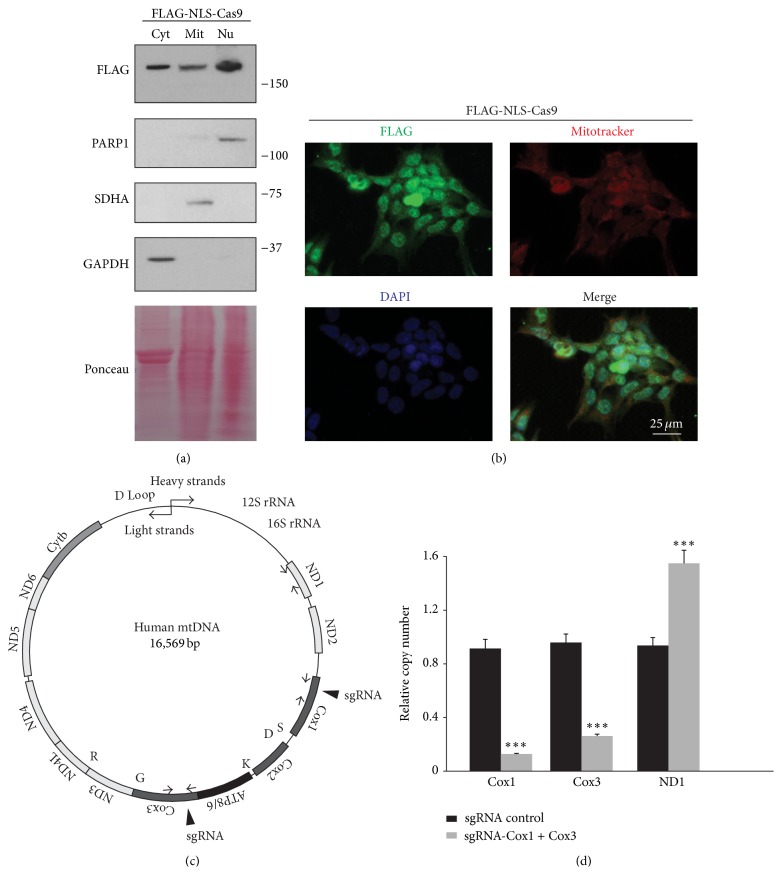
FLAG-NLS-Cas9 localizes to mitochondria. (a) Subcellular localization of FLAG-Cas9 assessed in the cytosolic (Cyt), mitochondrial (Mit), and nuclear (Nu) fractions of HEK-293T cells transfected with lentiCRISPR-sgRNA-eGFP#2 and monitored by Western blot. Glyceraldehyde 3-phosphate dehydrogenase (GAPDH) served as a cytosolic marker, poly(ADP-ribose) polymerase 1 (PARP1) served as a nuclear marker, and succinate dehydrogenase complex subunit A (SDHA) served as a mitochondrial marker. Ponceau staining of the blotted nitrocellulose membrane was presented in the bottom lane to visualize relative protein loading amounts. (b) Immunofluorescence images of FLAG-NLS-Cas9 demonstrating its localization to nucleus, cytoplasm, and mitochondria. HEK-293T cells were immunostained with mouse antibody to FLAG (green) 24 hours following transient transfection with FLAG-NLS-Cas9 construct. Mitochondria or nucleus was stained with mitotracker Red or DAPI, respectively. (c) Illustration of human mitochondrial DNA (mtDNA). Simplified view of mtDNA is presented to depict regions encoding peptides or tRNA for indicated amino acids. Filled triangles indicate sites targeted by gRNAs against Cox1 or Cox3. Arrows indicate primer annealing sites used for real-time PCR of Cox1 or Cox3 regions. (d) Quantification of copy numbers for Cox1, Cox3, and ND1 regions of mtDNA extracted from HEK-293T cells transiently transfected with lentiCRISPR-sgRNA-Cox1 and lentiCRISPR-sgRNA-Cox3 or lentiCRISPR-sgRNA-eGFP#2 as a control, determined by real-time quantitative PCR using primers listed in Table 2. GAPDH was used as an internal loading control (*n* = 3 per group). Quantified data (d) are expressed as mean ± s.e.m., ^*∗∗∗*^ *P* < 0.001, unpaired two-tailed Student's* t*-test.

The Clustered Regularly Interspaced Short Palindromic Repeats (CRISPR) defense system in bacteria has been successfully applied to edit specific genomic loci in many species [11]. CRISPR-associated (Cas) genes together with a single guide RNA (sgRNA) enable sequence-specific cleavage and editing of DNA. The currently available CRISPR/Cas9 system uses nuclear localization sequence- (NLS-) tagged SpCas9 to facilitate nuclear transfer of SpCas9, where genome editing should occur [12]. However, the precise subcellular distribution has not been examined. To determine in which subcellular compartment overexpressed FLAG-tagged SpCas9 is present, we performed subcellular fractionation into cytoplasmic, mitochondrial, and nuclear fractions for HEK-293T cells transiently transfected with lentiCRISPR-sgRNA-eGFP#2 construct, which should express FLAG-tagged Cas9 and sgRNA against eGFP. As expected, FLAG-Cas9 was detected in the nuclear fraction (Figure 1(a)). However, overexpression of FLAG-Cas9 showed substantial distribution to the other compartments, cytoplasm and mitochondria (Figure 1(a)). Consistent with the results from subcellular fractionation, immunofluorescence for overexpressed FLAG-Cas9 demonstrates its colocalization with mitotracker red-labeled mitochondria and DAPI-stained nucleus (Figure 1(a)).

### 3.2. CRISPR/Cas9-Mediated mtDNA Cleavage

Based upon this result showing FLAG-Cas9 distribution to mitochondria, we hypothesized that the CRISPR/Cas9 system could be implemented to edit mitochondrial DNA (mtDNA) when combined with sgRNA targeting specific loci of mtDNA. To test this possibility, we created several sgRNA targeting mtDNA regions encoding peptides (Cox1 and Cox3) that function with oxidative phosphorylation complexes (Figure 1(c)). Oligonucleotides designed to target each mtDNA region (Table 1) were cloned into lentiCRISPR plasmid, which can express sgRNA under the U6 promoter and SpCas9 under the EFS promoter. To examine whether the CRISPR/Cas9 system can cleave mtDNA specifically as it does for genomic DNA, we transfected HEK-293T cells with both lentiCRISPR-sgRNA-Cox1 and lentiCRISPR-sgRNA-Cox3. These constructs should cleave mtDNA and generate mtDNA fragments. LentiCRISPR-sgRNA-eGFP#2 was used as a control. Specific cleavage of mtDNA was evaluated using primers flanking the cleavage sites (primers for Cox1 and Cox3; please refer to Figure 1(b) and Table 2 for detailed information on primers and annealing loci). According to real-time PCR, there was an almost 90% reduction in intact mtDNA for the Cox1 locus in HEK-293T cells transfected with both lentiCRISPR-sgRNA-Cox1 and lentiCRISPR-sgRNA-Cox3 compared to control cells transfected with lentiCRISPR-sgRNA-eGFP#2 plasmid (Figure 1(d)). We also observed 80% cleavage for Cox3 locus with lentiCRISPR-sgRNA-Cox1 and lentiCRISPR-sgRNA-Cox3 transfection (Figure 1(d)). The noncleaved site (ND1 locus) with our lentiCRISPR constructs was also monitored using specific primers (Table 2). Interestingly, the mtDNA copy number as determined by ND1 site RTQ PCR was increased by approximately 50% (Figure 1(d)). These results indicate that FLAG-Cas9 can actually move into the matrix of mitochondria, and, when combined with sgRNA targeting mtDNA, it cleaves mtDNA in a site-specific manner.

### 3.3. Alteration of Mitochondrial Proteins Induced by CRISPR/Cas9-Mediated mtDNA Cleavage

**Figure 2:**
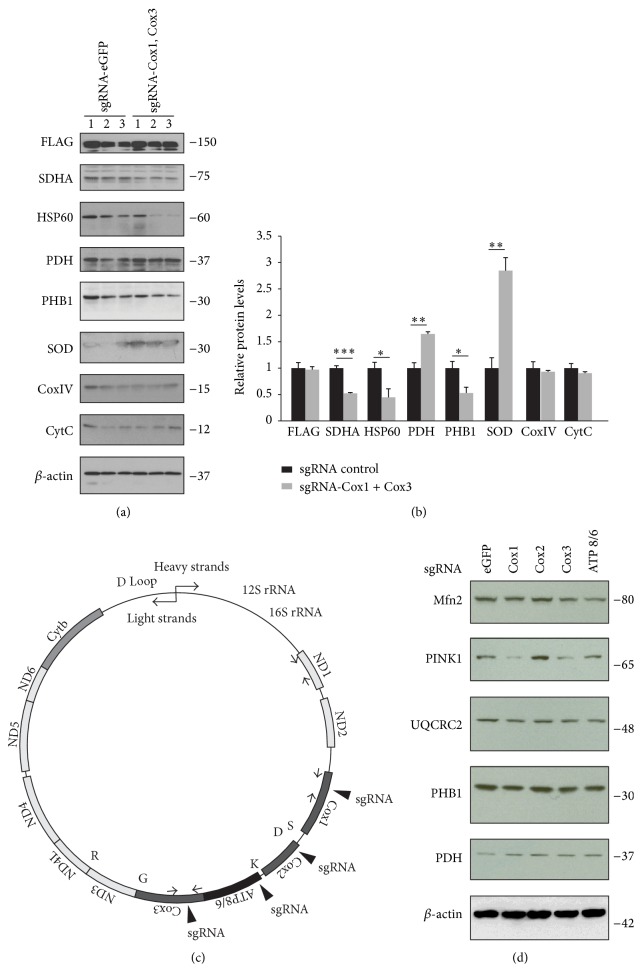
Alterations in mitochondria-associated proteins following CRISPR/Cas9-mediated mtDNA editing. (a) Mitochondrial proteins in HEK-293T cells after CRISPR/Cas9-mediated cleavage of mtDNA at Cox1 and Cox3 loci as determined by Western blots using indicated antibodies. *β*-actin was used as a loading control. (b) Quantification of mitochondria proteins in HEK-293T cells transfected with lentiCRISPR-sgRNA-Cox1 + Cox3 or lentiCRISPR-sgRNA-eGFP#2 control as shown in panel (a) normalized to *β*-actin. (c) Illustration of human mtDNA. Specific loci targeted by lentiCRISPR-sgRNAs (Cox1, Cox2, Cox3, and ATP8/6) are indicated with filled triangles. (d) Representative Western blots showing differential alteration of mitochondrial proteins following cleavage of specific mtDNA loci mediated by indicated sgRNAs in HEK-293T cells. Quantified data (b) are expressed as mean ± s.e.m., ^*∗*^ *P* < 0.05, ^*∗∗*^ *P* < 0.01, and ^*∗∗∗*^ *P* < 0.001, unpaired two-tailed Student's* t*-test.

Next, we examined alterations in mitochondria-associated proteins as an indication of disturbance of mitochondria that could be induced by CRISPR/Cas9-mediated mtDNA cleavage. When mtDNA was truncated at the Cox1 and Cox3 loci by CRISPR/Cas9, transcription of mtDNA heavy strands and thus expression of downstream genes from Cox1 locus could be affected (Figure 1(b)). When we monitored several mitochondrial marker proteins, there were alterations in several proteins: the levels of SDHA, heat shock protein 60 (HSP60), and prohibitin 1 (PHB1) decreased, whereas pyruvate dehydrogenase (PDH) and superoxide dismutase 1 (SOD1) levels increased (Figures 2(a) and 2(b)). However, no significant changes were observed in CoxIV or Cytochrome C (CytC) (Figures 2(a) and 2(b)).

Due to the distribution of tRNA and peptide-encoding regions and bidirectional transcriptions of mtDNA, cleavage at different loci of mtDNA could exert differential outcomes (Figure 2(c)). In this regard, we also examined whether cleavage of different mtDNA single loci led to differential regulation of mitochondria-associated proteins. HEK-293T cells were transfected with sgCox1, sgCox2, sgCox3, or sgATPase8/6. Each sgRNA transfection exerted differential effects on mitochondria-associated proteins. Cleavage of Cox1 or Cox3 or ATP8/6 regions led to slight reduction in ubiquinol-cytochrome c reductase core protein 2 (UQCRC2) and PHB1, whereas PDH level increased slightly (Figure 2(d)). Interestingly, mitochondria-associated PD gene PTEN-induced putative kinase 1 (PINK1) level decreased when Cox1 or Cox3 loci were cleaved, while cleavage of Cox2 regions resulted in an increase in PINK1 level (Figure 2(d)). Mitofusin-2 (Mfn2) for mitochondrial fission decreased when Cox1, Cox3, or ATP8/6 loci were cleaved by the CRISPR/Cas9 system (Figure 2(d)). Together, these results indicate that CRISPR/Cas9-mediated mtDNA cleavage results in an alteration of mitochondrial protein homeostasis. Moreover, depending on the loci of mtDNA cleavage, the pattern of dysregulation of mitochondrial proteins in early response to mtDNA cleavage differs. Changes in Mfn2 or PINK1 suggest a potential compensatory response for mitochondria quality control in cells following mtDNA damage.

### 3.4. Mitochondria-Targeted Cas9

**Figure 3:**
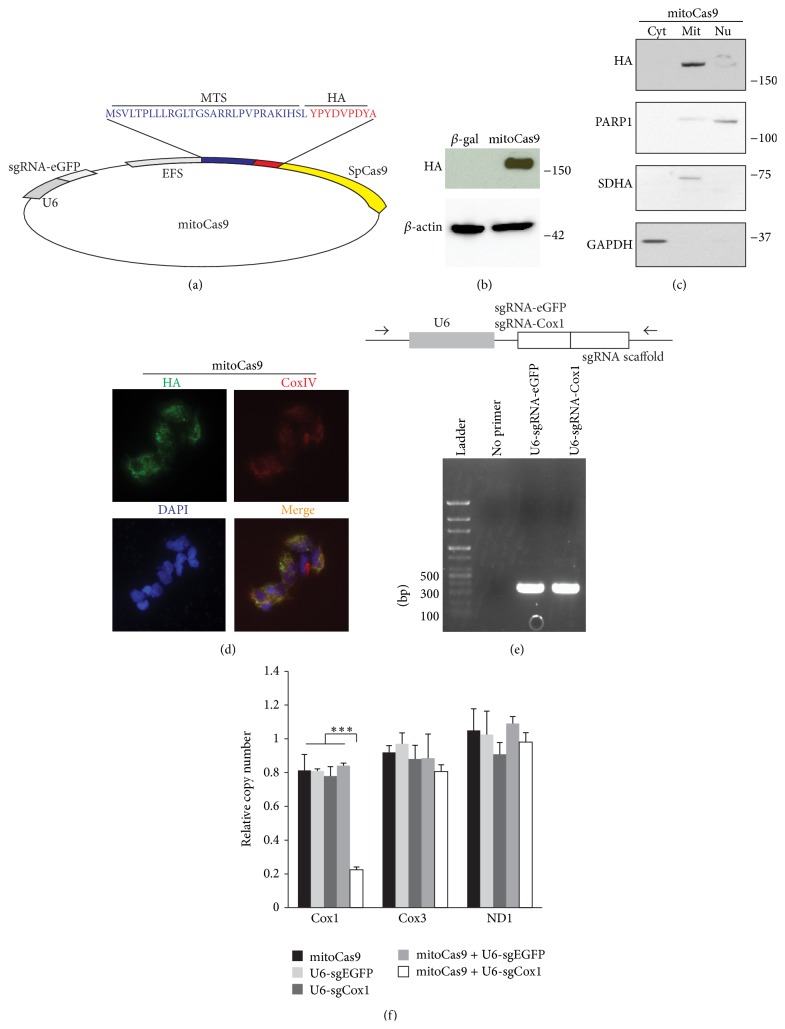
Construction of mitochondria-targeted MTS-HA-Cas9. (a) Schematic illustration of mitochondria-targeting Cas9 (mitoCas9). Mitochondria-targeting sequence (MTS) and HA tag information are presented. (b) Expression of mitoCas9 in HEK-293T cells transiently transfected with MTS-HA-Cas9 construct determined by Western blots using HA antibody. *β*-actin was used as a loading control. (c) Subcellular localization of MTS-HA-Cas9 assessed in the cytosolic (Cyt), mitochondrial (Mit), and nuclear (Nu) fractions of HEK-293T cells transfected with lentiCRISPR-sgRNA-eGFP#2 and monitored by Western blot. GAPDH served as a cytosolic marker, PARP1 served as a nuclear marker, and SDHA served as a mitochondrial marker. (d) Representative immunofluorescence microscopic image of MTS-HA-Cas9 (HA, green) and CoxIV (red) subcellular distributions in HEK-293T cells transfected with MTS-HA-Cas9 construct. The merged image (yellow, right panel) shows colocalization of mitoCas9 and CoxIV. (e) Representative gel images of the PCR product of hU6-sgRMA-eGFP and hU6-sgRNA-Cox1 which were purified by gel extraction (bottom panel). Schematics of primers and lentiCRISPR-sgRNA templates used to amplify U6 promoter and respective sgRNA components for transfection (upper panel). (f) Quantification of copy numbers for Cox1, Cox3, and ND1 regions of mtDNA extracted from HEK-293T cells transiently transfected with indicated constructs (mitoCas9 is a plasmid, while U6-sgRNAs are PCR product.), determined by real-time quantitative PCR using primers listed in Table 2. GAPDH was used as an internal loading control (*n* = 3 per group). Quantified data (b) are expressed as mean ± s.e.m., ^*∗∗∗*^ *P* < 0.001, analysis of variance (ANOVA) test followed by Student-Newman-Keuls post hoc analysis.

Although we demonstrated that FLAG-Cas9 with NLS peptide can effectively localize to mitochondria for functional editing of mtDNA, Cas9, which can specifically localize to mitochondria, is required for safe application for mtDNA editing without affecting genomic DNA. Therefore, we aimed at creating a new version of Cas9 that can specifically target mtDNA. By removing FLAG and NLS sequences from lentiCRISPR-sgRNA-eGFP#2 and adding mitochondrial targeting sequence (MTS) and HA tag in the N-terminus of SpCas9, we synthesized mitochondria-targeting Cas9 (mitoCas9) (Figure 3(a)). MTS of cytochrome C (mitochondria matrix protein) was used to direct mitoCas9 into the matrix of mitochondria so that it can interact with mtDNA. MitoCas9 was expressed and detected with HA-specific antibody at the expected molecular weight (Figure 3(b)), and subcellular fractionation showed that mitoCas9 mainly localized to mitochondria (Figure 3(c)). In addition, immunofluorescence confirmed colocalization of HA-tagged mitoCas9 with mitochondria marker protein CoxIV (Figure 3(d)).

### 3.5. Mitochondrial DNA Editing by mitoCas9

To determine whether this new version of Cas9 is functional for mtDNA editing, we transfected HEK-293T cells with both mitoCas9 and DNA expressing sgRNA for Cox1. For this application, we PCR-amplified hU6 promoter and sgRNA-Cox1 regions from lentiCRISPR-sgRNA-Cox1 plasmid (Figure 3(e)). U6-sgRNA-eGFP was also PCR-amplified to be used as control (Figure 3(e)). As expected, mitoCas9 and hU6-sgRNA-Cox1 robustly cleaved the targeted region of the mtDNA as determined by real-time quantitative PCR only at Cox1 targeted locus (Figure 3(f)). Taken together, our results demonstrate that mitochondria-targeting mitoCas9 can be applied to edit mtDNA in conjunction with custom-designed sgRNAs.

### 3.6. Mitochondrial Dysfunction Induced by mitoCas9-Mediated mtDNA Damage

**Figure 4:**
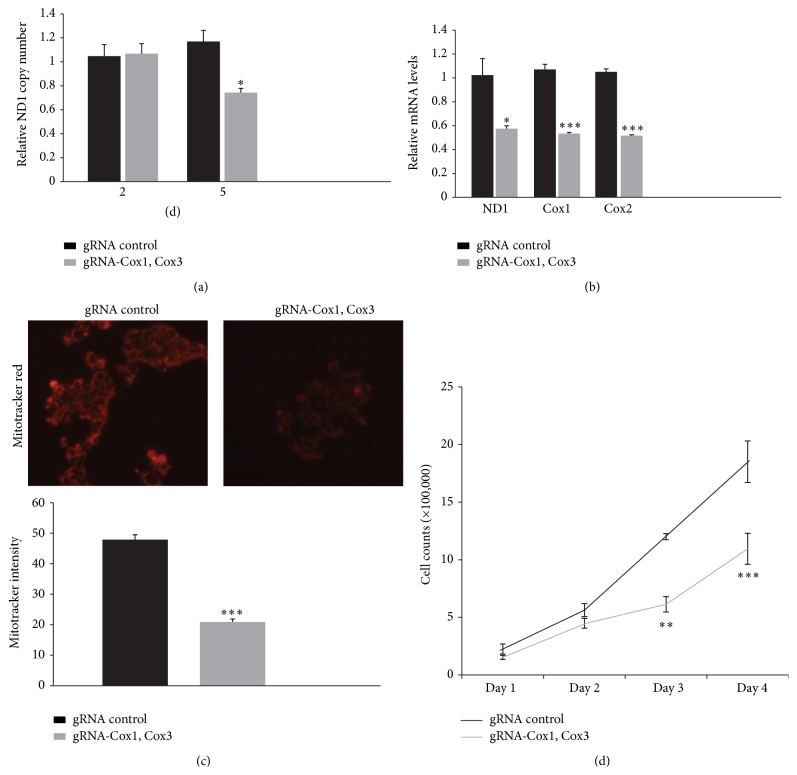
MitoCas9-induced mtDNA damage leads to mitochondria dysfunction. (a) Quantification of copy numbers for ND1 region of mtDNA extracted from HEK-293T cells transiently transfected with indicated constructs (gRNA control, mitoCas9 and U6-sgRNA to eGFP; gRNA Cox1, Cox3, mitoCas9, and U6-sgRNA to Cox1 and Cox3), determined by real-time quantitative PCR using primers listed in Table 2 at the indicated time points (2 days and 5 days after transfection). GAPDH was used as an internal loading control (*n* = 3 per group). (b) Quantification of messenger RNAs for ND1, Cox1, and Cox2 genes in HEK-293T cells 5 days following transient transfection with lentiCRISPR-sgRNA-Cox1 + Cox3 or lentiCRISPR-sgRNA-eGFP#2 control determined by real-time PCR and normalized to GAPDH (*n* = 3 per group). (c) Representative images of mitotracker Red staining for functional mitochondria in HEK-293T cells transfected with the indicated constructs (upper panel). Quantification of relative mitotracker Red staining intensities in two groups was shown in the bottom panel (*n* = 50 mitochondria from three independent cultures per group). (d) Cell counts demonstrating proliferation rate of the HEK-293T cells following cleavage of specific mtDNA loci (*n* = 6 individual measurements per group). Days indicate the time intervals proceeded after cell seeding following 5 days of transient transfectin. Quantified data are expressed as mean ± s.e.m., ^*∗*^ *P* < 0.05, ^*∗∗*^ *P* < 0.01, and ^*∗∗∗*^ *P* < 0.001, unpaired two-tailed Student's* t*-test.

Mitochondria targeting of certain restriction enzymes such as EcoRI or PstI has been employed to destroy mitochondrial DNA [13, 14] together with chemical induction of mtDNA elimination. To determine whether mitoCas9-mediated site-specific mtDNA cleavage leads to functional disruption of mitochondria, we first examined mtDNA copy number and several mitochondrial gene expressions. 5 days following transient transfection of HEK-293T cells with mitoCas9 and sgRNA targeting Cox1 and Cox3 regions, real-time PCR quantification of mtDNA copy number was performed using ND1 primers. Cell division for 5 days with site-specific mitoCas9 cleavage at Cox1 and Cox3 regions leads to approximate 30% reduction of mtDNA copy numbers (Figure 4(a)). Consistent with the fact that mtDNA was cleaved and steady state mtDNA copy number was reduced by CRISPR/Cas9 system, the amounts of messenger RNAs encoding mitochondrial ND1, Cox1, and Cox2 genes were reduced by about 50% compared to those of sgRNA control HEK-293T cells (Figure 4(b)).

Studies have shown that mitochondrial dysfunction induced by mtDNA damage results in alterations on mitochondrial membrane potential and cell growth capacity. To assess whether mitoCas9 medicated mtDNA cleavage and mtDNA reduction affect mitochondrial and cellular physiology, we measured mitochondria membrane potential by using mitotracker Red which stains functional mitochondria in a membrane potential sensitive manner. Consistent with the notion that mitochondrial gene expression and proteomic homeostasis are disturbed by mitoCas9-mediated mtDNA damage, mitochondrial membrane potential was impaired for HEK-293T cells transfected with mitoCas9 and sgRNA targeting Cox1 and Cox3 compared to that of control cells (Figure 4(c)). Cell proliferation rates were also examined for these mitoCas9 induced HEK-293T cells of mitochondrial dysfunction. When monitored for four consecutive days after seeding, HEK-293T cells transfected with sgRNA to Cox1 and Cox3 exhibit decelerated growth rate compared to control cell line (Figure 4(d)).

Taken together, our results demonstrate that mitoCas9-induced mtDNA damage leads to functional defects of mitochondria and ultimately causes reduction in cell proliferation capacity.

## 4. Discussion

We discovered that CRISPR/Cas9-mediated genome editing can be successfully employed to manipulate the mitochondrial genome. This approach still needs further study to understand how gRNA can be translocated into the mitochondria matrix together with mitochondria-localizing Cas9. It is interesting to note their tropism to mitochondria even with artificially added nuclear localizing sequence. Given the wide application of CRISPR/Cas9 in genome manipulation, researchers should be careful when studying mitochondria function because Cas9 can localize to mitochondria for unwanted mtDNA editing depending on the sgRNA sequences used for the study. Pseudogenes for genomic Cox1 and Cox3 are present and they may have been affected with expression of FLAG-NLS-Cas9 targeting mtCox1 and mtCox3 regions.

One group has reported generation and characterization of knock-in mice expressing Cas9 [15]. This transgenic line was successfully used to knockout genes of interests in brains, lungs, and other tissues depending on the injection sites of gRNA-expressing viral vectors. Although further characterization of Cas9 subcellular localization is required* in vivo*, our data suggest that Cas9-expressing transgenic mice can be manipulated to affect the mitochondrial genome with viral injection of gRNA targeting specific mtDNA loci.

Mitochondrial sequence-specific cleavage including truncation is possible with the CRISPR/Cas9 system. This approach could be applied to study functional roles of mitochondria DNA damage repair protein complexes. DNA damage repair machinery has been reported in mitochondria although its repair efficiency is known to be limited compared to that of nuclear genomic DNA repair [16]. Despite identification of several mitochondrially localized repair enzymes, their actual roles in maintenance of mitochondrial DNA remain to be elucidated. In this respect, our discovery of the role of CRISPR/Cas9 in mtDNA editing provides a tool to determine how mtDNA cleavage can be handled and repaired. According to our results, once cleavage on specific loci of mtDNA and truncation is achieved by the CRISPR/Cas9 system, there seems to be compensatory amplification of intact circular mtDNA as determined by an increase in the copy number of uncut loci. During 2-3 days of CRISPR/Cas9-mediated cleavage, the cleaved linear mtDNA appears to be present. To understand the repair process or clearance of cleaved mtDNA, longer time points after expression of CRISPR/Cas9 should be examined.

Mitochondria-targeting Cas9 combined with mtDNA-specific sgRNA provides an opportunity to generate mutant mtDNA. Design of a conventional method to study mutant mtDNA in cells has been challenging [17]. By controlling the expression of mitochondrial Cas9 and selecting single cell lines with desired mutations, it is possible to create a cell model that contains normal and dysfunctional mitochondria in order to study the role of specific mtDNA mutations in related human diseases.

We also showed that the CRISPR/Cas9 system-mediated cleavage of different loci of mtDNA initiates differential alterations of mitochondria-associated proteins. Mitochondrial transcription involves polycistronic expression of peptides, tRNA, and rRNA from several long RNAs, either heavy strands or light strands. In this regard, it is not surprising that there is differential disruption of the mitochondrial transcriptome depending on the cleavage loci. The alterations in mitochondria-associated proteins assessed in this study may reflect complex characteristics of the mitochondrial proteome and response to mtDNA damage. Time-course monitoring of the mitochondrial proteome after CRISPR/Cas9-mediated mtDNA cleavage may provide some clues on how mitochondrial function is regulated against mtDNA damage.

Successful demonstration of mtDNA editing by the CRISPR/Cas9 system suggests its therapeutic potential. Mitochondria-targeting TALEN (mitoTALEN) has been shown to selectively remove mutation-harboring mtDNA [10]. In a similar fashion, with corresponding sgRNA design targeting frequent mtDNA mutations, CRISPR/Cas9 could be used to treat cells harboring mutant mtDNAs. Particularly, mitoCas9 can reduce nonspecific targeting of genomic DNA, thus alleviating any potential side effects during therapeutic application compared to that with regular Cas9, which demonstrates nonspecific subcellular distribution.

## 5. Conclusions

In this brief report, we first establish a proof of concept model which demonstrates that CRISPR/Cas9 can be employed to target specific loci of mitochondrial genome. We showed regular Cas9 nuclear enzyme has a tendency to localize to mitochondria and somehow gRNA can be transported together with mitochondrial matrix for its function. Given the vast amount of CRISPR/Cas9 tools available, our discovery of Cas9 editing of mitochondrial genome will provide a new avenue of its application to mitochondria study. Additionally, we also provided reengineering of the regular Cas9 to restrict its localization to mitochondria matrix. This mitochondria-targeting Cas9 (mitoCas9) is functional and cleaves mtDNA at the site guided by specific sgRNA. Viral production or animal model generation of mitoCas9 would be beneficial to expand these findings to* in vivo* studies of mitochondria.

## Figures and Tables

**Table 1:** Oligonucleotides used for gRNA construction.

mtDNA	Forward	Reverse
Cox1	CACCGGGCCCAGCTCGGCTCGAATA	AAACTATTCGAGCCGAGCTGGGCCC
Cox2	CACCGTATGAGGGCGTGATCATGAA	AAACTTCATGATCACGCCCTCATAC
Cox3	CACCGGCCTAGTATGAGGAGCGTTA	AAACTAACGCTCCTCATACTAGGCC
ATP8/6	CACCGTCGTCCTTTAGTGTTGTGTA	AAACTACACAACACTAAAGGACGAC

**Table 2:** Real-time PCR primers.

Gene	Forward	Reverse
Cox1	CGCCGACCGTTGACTATTCT	GGGGGCACCGATTATTAGGG
Cox2	TTCATGATCACGCCCTCATA	TAAAGGATGCGTAGGGATGG
Cox3	CAGCCCATGACCCCTAACAG	TGTGGTGGCCTTGGTATGTG
ND1	TCTCACCATCGCTCTTCTAC	TTGGTCTCTGCTAGTGTGGA
GAPDH	CATGTGCAAGGCCGGCTTCG	CTGGGTCATCTTCTCGCGGT
GAPDH for cDNA	AAACCCATCACCATCTTCCAG	AGGGGCCATCCACAGTCTTCT

Forward and reverse primers are designed to anneal upstream and downstream regions flanking gRNA target sites for each mtDNA locus or specific genes.

## References

[1] Bratic I., Trifunovic A. (2010). Mitochondrial energy metabolism and ageing. *Biochimica et Biophysica Acta*.

[2] Carafoli E. (2003). Historical review: mitochondria and calcium: ups and downs of an unusual relationship. *Trends in Biochemical Sciences*.

[3] Tait S. W. G., Green D. R. (2010). Mitochondria and cell death: outer membrane permeabilization and beyond. *Nature Reviews Molecular Cell Biology*.

[4] Taanman J.-W. (1999). The mitochondrial genome: structure, transcription, translation and replication. *Biochimica et Biophysica Acta—Bioenergetics*.

[5] Filosto M., Scarpelli M., Cotelli M. S. (2011). The role of mitochondria in neurodegenerative diseases. *Journal of Neurology*.

[6] Johri A., Beal M. F. (2012). Mitochondrial dysfunction in neurodegenerative diseases. *The Journal of Pharmacology and Experimental Therapeutics*.

[7] Moore D. J., West A. B., Dawson V. L., Dawson T. M. (2005). Molecular pathophysiology of Parkinson's disease. *Annual Review of Neuroscience*.

[8] Deas E., Wood N. W., Plun-Favreau H. (2011). Mitophagy and Parkinson's disease: the PINK1-parkin link. *Biochimica et Biophysica Acta*.

[9] Martinez T. N., Greenamyre J. T. (2012). Toxin models of mitochondrial dysfunction in Parkinson's disease. *Antioxidants and Redox Signaling*.

[10] Bacman S. R., Williams S. L., Pinto M., Peralta S., Moraes C. T. (2013). Specific elimination of mutant mitochondrial genomes in patient-derived cells by mitoTALENs. *Nature Medicine*.

[11] Hsu P. D., Lander E. S., Zhang F. (2014). Development and applications of CRISPR-Cas9 for genome engineering. *Cell*.

[12] Cong L., Ran F. A., Cox D. (2013). Multiplex genome engineering using CRISPR/Cas systems. *Science*.

[13] Kukat A., Kukat C., Brocher J. (2008). Generation of *ρ*
^0^ cells utilizing a mitochondrially targeted restriction endonuclease and comparative analyses. *Nucleic Acids Research*.

[14] Srivastava S., Moraes C. T. (2005). Double-strand breaks of mouse muscle mtDNA promote large deletions similar to multiple mtDNA deletions in humans. *Human Molecular Genetics*.

[15] Platt R. J., Chen S., Zhou Y. (2014). CRISPR-Cas9 knockin mice for genome editing and cancer modeling. *Cell*.

[16] Larsen N. B., Rasmussen M., Rasmussen L. J. (2005). Nuclear and mitochondrial DNA repair: similar pathways?. *Mitochondrion*.

[17] Chandel N. S., Schumacker P. T. (1999). Cells depleted of mitochondrial DNA (*ρ*0) yield insight into physiological mechanisms. *FEBS Letters*.

